# Multiple active site residues are important for photochemical efficiency in the light-activated enzyme protochlorophyllide oxidoreductase (POR)^☆^

**DOI:** 10.1016/j.jphotobiol.2016.05.029

**Published:** 2016-08

**Authors:** Binuraj R.K. Menon, Samantha J.O. Hardman, Nigel S. Scrutton, Derren J. Heyes

**Affiliations:** a Centre for Synthetic Biology of Fine and Speciality Chemicals, Manchester Institute of Biotechnology, School of Chemistry, The University of Manchester, Manchester, M1 7DN, UK

**Keywords:** Protochlorophyllide oxidoreductase (POR), Light activation, Photochemistry, Site-directed mutagenesis, Enzyme catalysis

## Abstract

Protochlorophyllide oxidoreductase (POR) catalyzes the light-driven reduction of protochlorophyllide (Pchlide), an essential, regulatory step in chlorophyll biosynthesis. The unique requirement of the enzyme for light has provided the opportunity to investigate how light energy can be harnessed to power biological catalysis and enzyme dynamics. Excited state interactions between the Pchlide molecule and the protein are known to drive the subsequent reaction chemistry. However, the structural features of POR and active site residues that are important for photochemistry and catalysis are currently unknown, because there is no crystal structure for POR. Here, we have used static and time-resolved spectroscopic measurements of a number of active site variants to study the role of a number of residues, which are located in the proposed NADPH/Pchlide binding site based on previous homology models, in the reaction mechanism of POR. Our findings, which are interpreted in the context of a new improved structural model, have identified several residues that are predicted to interact with the coenzyme or substrate. Several of the POR variants have a profound effect on the photochemistry, suggesting that multiple residues are important in stabilizing the excited state required for catalysis. Our work offers insight into how the POR active site geometry is finely tuned by multiple active site residues to support enzyme-mediated photochemistry and reduction of Pchlide, both of which are crucial to the existence of life on Earth.

## Highlights

• Identified several active site residues that can interact with coenzyme/substrate
• Multiple residues are important in excited state POR–protochlorophyllide interactions.
• New structural model for *T. elongatus* POR to rationalize mutagenesis outcomes
• POR active site geometry is finely-tuned to support photochemistry.

## Graphical abstract

**Figure:**
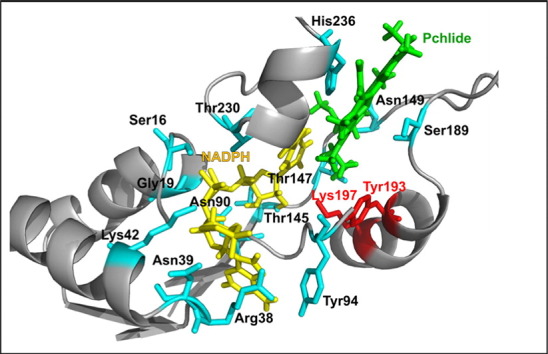

## 1 Introduction

**Fig. 1:**
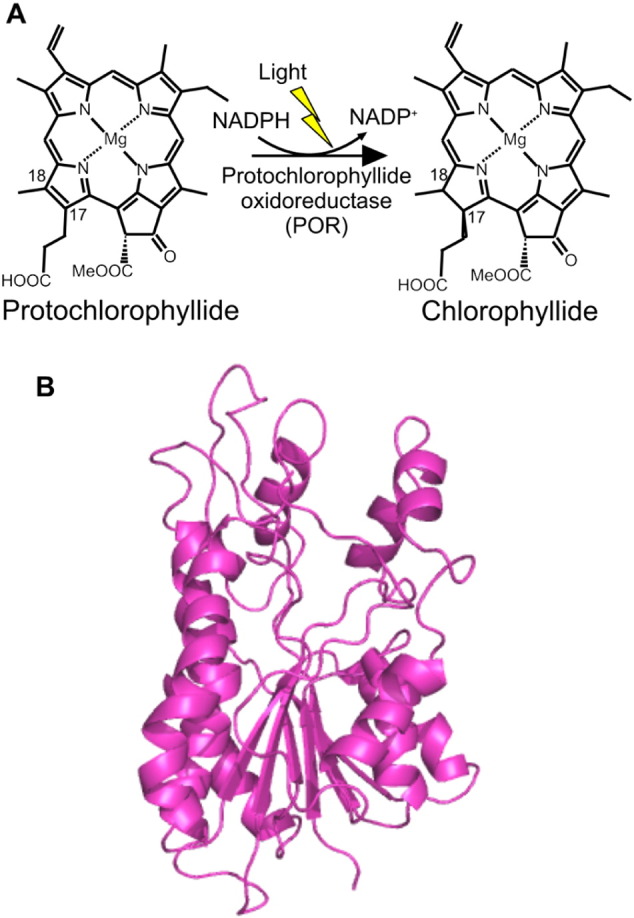
The light-activated reduction of Pchlide catalyzed by POR. A, *trans* addition of hydrogen across the C_17_=C_18_ carbon double bond of Pchlide to form Chlide in the chlorophyll biosynthesis pathway is catalyzed by protochlorophyllide oxidoreductase (POR). B, the three-dimensional homology model of POR from *Synechocystis*, based on the crystal structure of 7α-hydroxysteriod dehydrogenase as a structural template [27].

The reduction of protochlorophyllide (Pchlide) to chlorophyllide, catalyzed by the enzyme protochlorophyllide oxidoreductase (POR), is the key light-driven reaction that leads to a profound transformation in plant development [1], [2], [3], [4], [5]. In non-flowering plants, algae and cyanobacteria there is also a light-independent Pchlide reductase, consisting of 3 separate subunits, which can catalyze this same penultimate step in the chlorophyll biosynthetic pathway [6], [7]. In light-dependent POR the reaction proceeds via a highly endergonic light-driven hydride transfer from the NADPH coenzyme to the C17 position of the Pchlide molecule followed by an exergonic thermally activated proton transfer from a conserved Tyr residue to the C18 position of Pchlide (Fig. 1A) [8], [9], [10], [11], [12]. Recent advances in our understanding of the POR reaction mechanism that illustrate POR is an important model for studying light-activated enzyme dynamics and how light energy can be harnessed to power biological catalysis [13], [14], [15], [16], [17], [18].

PORs originating from a variety of photosynthetic organisms, including cyanobacteria and higher plants, have been studied in detail using ultrafast and cryogenic spectroscopic techniques [8], [16], [18], [19], [20], [21]. Excited state interactions between the Pchlide molecule and active site residues in the enzyme are proposed to result in a reactive charge-separated state that facilitates the sequential hydride and proton transfer reactions on a microsecond timescale [7], [15], [18], [22]. The light-driven hydride transfer step is coupled to enzyme motions within the lifetime of the Pchlide excited state and is followed by the proton transfer step, which is reliant on solvent dynamics and an extended network of molecular motions [22]. The catalytic cycle of POR also comprises a series of conformational changes that are associated with ordered substrate binding and product release steps to form a reactive active site conformation [7], [23], [24]. However, despite a detailed molecular understanding of the catalytic cycle from femtoseconds to seconds the structural features of POR, and active site residues that contribute to light activation and the reaction dynamics, is currently unknown due to the lack of a crystal structure.

**Fig. 2:**
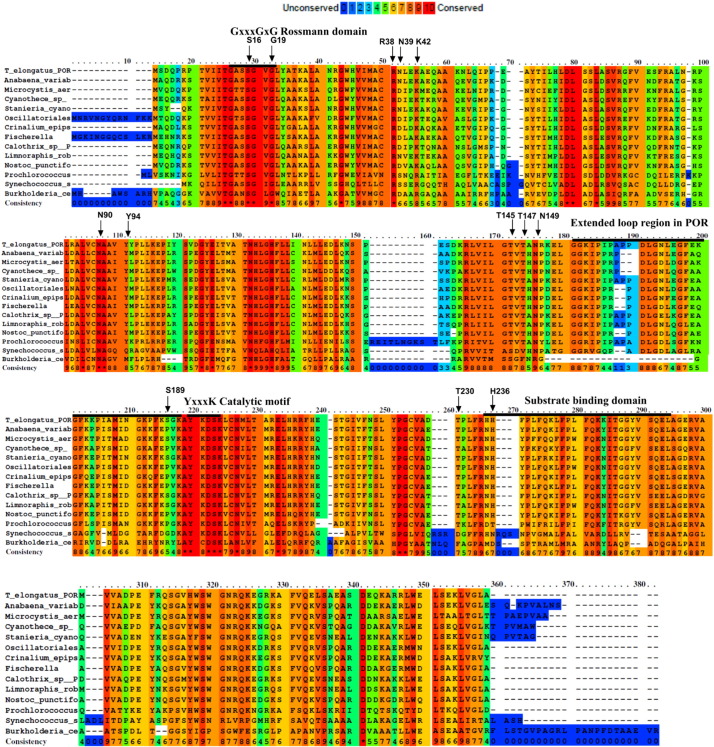
The sequence alignment and conservation of amino acid among homologous POR enzymes. The protein sequence of *T. elongatus* POR is aligned with different homologous POR enzymes. The amino acid residues that were targeted in this study are indicated by arrows. The conserved regions are indicated by black lines. PRALINE; protein multiple sequence alignment web server tool (by Centre for Integrative Bioinformatics, Vrije Universiteit Amsterdam, Netherlands) was used for the alignment of protein sequences.

Sequence similarities with other short-chain dehydrogenase/reductase (SDR) enzymes identifies POR as a member of the classical SDR family of enzymes. The conserved glycine rich-Rossmann dinucleotide-binding domain (GxxxGxG) and the catalytic YxxxK motifs of POR have suggested a similar catalytic mechanism in POR and other SDR enzymes [25], [26], [27]. Consequently, although the overall sequence homology and pair wise identity between POR and any individual SDR enzyme is below the requirement to select an empirical structural template, different homology models of POR have been proposed by using closely related SDR enzymes as the structural template [5], [27], [28], [29], [30]. These homology models were composed of 7 β-sheets surrounded by 9 α-helices (Fig. 1B). The extensions to some of the central helices provide the Pchlide binding cleft, where the YxxxK catalytic motif was included as part of an *α*-helix that results in residues Tyr193 and Lys197 interacting with the Pchlide molecule. A unique 33 amino acid residue insertion in POR was included as a loop region, as no corresponding structural regions were present in the template structures, and was predicted to be involved in establishing an oligomerisation domain for POR in barley [27], [31]. Based on these previous models we have now selected a number of active site variants to study the role of residues located in the proposed NADPH and Pchlide binding sites in the reaction mechanism of POR (Fig. 2). Detailed binding, multiple turnover and single turnover laser studies have shown that although many of the variants have reduced catalytic activity they all retain the ability to bind substrates, albeit with reduced affinity in some cases. Several of the active site variants show impaired photochemical behaviour, suggesting that multiple residues are likely to be involved in the formation of the excited state ‘reactive’ charge transfer state that is required prior to hydride transfer. In the ongoing absence of a crystal structure we have rationalized these findings by using multiple SDR enzymes as a structural template to produce an improved homology model of POR from *Thermosynechocystis elongatus*.

## 2 Materials and Methods

### 2.1 Homology Modelling

The homology model of POR from *Synechocystis* sp. [27] was used as the initial template for the development of a structural model of *Thermosynechococcus elongatus* (BP-1) POR, using SWISS-MODEL homology server [32]. The NADPH coenzyme and Pchlide substrate were docked in to the model with AutoDock and the ligand-docked structures were relaxed by means of MD simulations by using the Amber ff94 force field implemented in Gromacs 4.6.5. Prior to this, the base structure was corrected and topology files for Gromacs were generated by using the online tools from MDweb [33]. The quality of developed homology model was analyzed using QMEAN server [34]. After optimisation the final model showed a QMEAN score of 0.50 and Z score of 2.95. In addition, the SWISS-MODEL homology server was used to determine the 15 SDR enzymes of known structures in the PDB that share the highest sequence identity with *T. elongatus* POR (Tables S1 and S2, Fig. S1). Mustang-MR structural sieving server was used for the multiple structural alignments of these SDR enzymes with POR and to remove residues from the alignment that are below a threshold root mean square deviation (RMSD) of 1.8 Å (Fig. S2) [35]. A putative uncharacterized SDR protein (PDB id: 3RD5) that has the highest sequence identity with POR was used as the reference structure and the sieving procedure removed 68% of the 3RD5 residues at 1.8 Å sieving level using Mustang-MR sieving server. The unsieved structural regions of 3RD5 were mapped on to the homology model of *T. elongatus* POR by structural alignment to determine the regions that are conserved between SDR enzymes and POR and will therefore be modeled most accurately.

### 2.2 Sample Preparation and Site Directed Mutagenesis

All chemicals were obtained from Sigma-Aldrich. Recombinant POR from the thermophilic cyanobacteria *T. elongatus* BP-1 was overexpressed in *Escherichia coli* and purified as described previously [36]. Site-directed mutagenesis of the *por* gene was performed using the QuikChange kit (Stratagene) to mutate residues. The primers that were used (MWG Eurofins, Germany) are shown in the supplementary information (Table S3). The correct mutations were confirmed by DNA sequencing (MWG Eurofins, Germany) and variant protein was purified as described previously [36]. The Pchlide pigment was produced and purified as described previously [37].

### 2.3 Steady-state Activity and Substrate Binding Measurements

Steady-state activity measurements were carried out as described previously using a Cary 50 spectrophotometer (Agilent Technologies) [38]. The binding of NADPH coenzyme was monitored using fluorescence energy resonance transfer in a Cary Eclipse fluorimeter (Agilent Technologies) [38] and the binding of Pchlide was measured by following the red-shift in absorbance at 642 nm, essentially as described [24].

### 2.4 Laser Flash Photolysis

Absorption transients at 696 nm were measured at 298 K using an LKS-60 flash photolysis instrument (Applied Photophysics Ltd.) with the detection system (comprising probe light, first monochromator, sample, second monochromator and photomultiplier) at right angles to the incident laser beam. Dark assembled enzyme–NADPH–Pchlide ternary complex samples were excited in a cuvette of 1-cm pathlength as described previously [10] with a 6 ns laser pulse at 450 nm (~ 30 mJ) using an OPO of a Q-switched Nd-YAG laser (Brilliant B, Quantel). The reaction samples were prepared by taking 15 μM Pchlide and 250 μM NADPH in the presence of 60.0 μM enzyme for wild-type POR. Based on the dissociation constant for Pchlide an equal concentration of ternary complex was used for the variant enzymes. Hence, the reaction samples (1 ml) were prepared by using 60 μM enzyme concentrations for R38V, K42A, T147S and S189A T230S and 55 μM for S16C, G19A and T230S. Higher concentrations of enzymes were used for N39V (128 μM), N90A (106 μM), Y94F (78 μM), T145 A (160 μM), T230 A (78 μM), T230F (175 μM), T147F (74 μM), N149 V (100 μM) and H236A (71 μM) variant enzymes. Rate constants were measured from the average of at least five time dependent absorption measurements by fitting to a single exponential function.

### 2.5 Ultrafast Pump-Probe Transient Absorption Spectroscopy

A Ti:sapphire amplifier system (Spectra Physics Solstice Ace) produced 6 mJ of 800 nm pulses at 1 kHz with 100 fs pulse duration. A portion of the output of the amplifier was used to pump a Topas Prime OPA with associated NirUVis unit which was used to generate the pump beam centred at 450 nm, with FWHM of ca. 10 nm. A broad band ultrafast pump-probe transient absorbance spectrometer ‘Helios’ (Ultrafast systems LLC) was used to collect data (at random time points) from ~ 1 ps to 2.6 ns with a time resolution of around 0.2 ps. The probe beam consisted of a white light continuum generated in a sapphire crystal and absorbance changes were monitored between 475 and 725 nm. A broad band sub-nanosecond pump-probe transient absorbance spectrometer ‘Eos’ (Ultrafast systems LLC) was used to collect data (at random time points) up to 2 μs with a time resolution of around 0.5 ns. A 2 kHz white-light continuum fibre laser was used to generate the probe pulses and the delay between pump and probe was controlled electronically. For both sets of measurements samples were excited at 450 nm with 0.5 μJ power and a beam diameter of ~ 200 μm. Samples (1.5 ml) were flowed at a rate of approximately 30 ml/min through a 0.2 mm pathlength quartz cell (at room temperature) to ensure that a different area of the sample is excited with each pump laser pulse. Samples were measured upon until the proportion of Chlide in the reaction mixture became higher than 10%, which resulted in data collection times of < 5 min for the wild-type and in the range of 8–20 min for the variants. Samples were prepared in the dark containing 500 μM POR, 200 μM Pchlide and 4 mM NADPH in activity buffer with 10% glycerol, 0.1% 2-mercaptoethanol, and 0.5% Triton X-100.

### 2.6 Global Analysis

The datasets from the Helios and Eos measurements were merged by selecting data with the same (small) proportion of product present and scaling the Eos dataset by a fixed factor to match the intensity of the ground state bleach feature to that in the Helios data. The data were then analyzed globally using the open-source software Glotaran [39]. This procedure reduces the matrix of change in absorbance as a function of time and wavelength, to a model of one or more exponentially decaying time components, as described in the main manuscript [39], each with a corresponding difference spectrum (species associated difference spectra (SADS)). Errors quoted with the lifetime values are the standard errors calculated during the global analysis. The lifetimes quoted for the conversion between states also include contributions from the rates of ground state recovery through both radiative and non-radiative processes. For the analysis, the pre-excitation background was subtracted, and Helios datasets corrected for spectral chirp, and the datasets were fitted to a simple sequential model where one species converts to another, which then persists for the lifetime of the experiment.

## 3 Results

### 3.1 A New Structural Model for POR

**Fig. 3:**
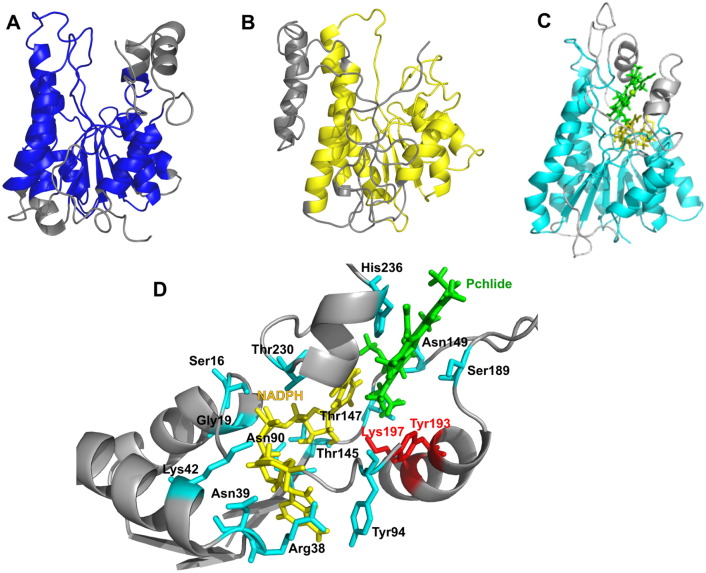
Structural models of POR from *Synechocystis* (A) [27], *Arabidopis thaliana* (B) [30] and from *T. elongatus* (C) described in the present work are shown for comparison. D, a close-up view of the active site illustrating the relative positions of the active site residues characterized in this study, which are shown in cyan, and the active site Tyr and Lys residues, which are shown in red as sticks. The protein backbone is represented as a ribbon, NADPH is shown in yellow and Pchlide is shown in green. In Fig. A, B and C, protein backbone is coloured in gray to indicate regions in the model which are not conserved.

**Fig. 4:**
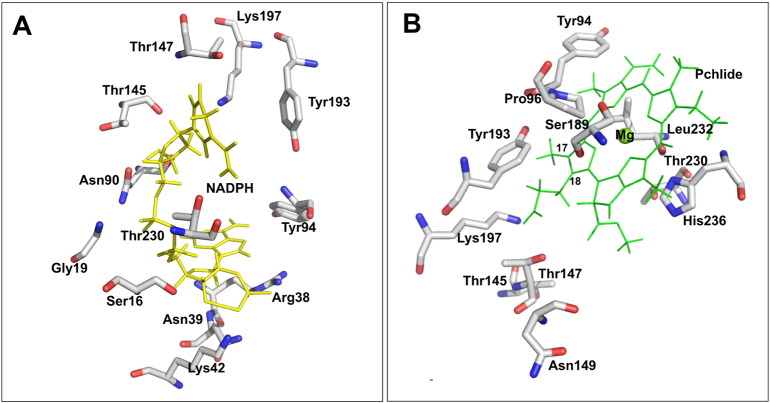
A close-up view of the NADPH (A) and Pchlide (B) binding site, showing potential active site amino acid residues that could interact with the NADH or Pchlide molecule based on the derived structural model of *T. elongatus* POR.

As there is currently no crystal structure available for the POR enzyme further insights into the role of putative active site residues in catalysis has been gained by producing a more accurate homology model for the enzyme. This has been achieved by selecting multiple templates of known PDB structures with a high sequence similarity with POR, rather than a single template, and by identifying the core regions conserved across all of the SDR enzymes (Figs. S1 and S2). A total of 15 SDR enzymes were selected as the targets of sequence and structure alignment with POR, where the identity of individual templates with POR varied between 19 and 30% (Table S2, Fig. S1) [32]. By using a structural sieving server, the highly conserved regions were mapped to the homology model to optimise the POR structure (Fig. 3). The overall structural model of *T. elongatus* POR is similar to previous models (Fig. 3A–C) [5], [27], [28], [29], [30] with the highly conserved Rossmann-fold region, surrounding β-sheets and α-helices and the catalytic tetrad residues, together with two insertions (residues 232 to 253 and residues 151 to 186) that are not present in other SDR enzymes and are therefore structurally less well-defined. Consequently, this refined model of POR provides important insights about the potential role of individual residues in catalysis. Many of the residues selected for mutagenesis are in close enough proximity to interact with the NADPH coenzyme. Ser16 and Gly19 are part of the highly conserved GxxxGxG motif, whereas Arg38, Asn39 and Lys42 are located close to the 2′-phosphate of the adenosine ribose group and Asn90 is located near to the adenine moiety of NADPH (Figs. 3D, 4A). Other residues, such as Tyr94, Thr145, Thr147, Asn149, Ser189, Thr230 and His236 are also positioned in the active site, close to both Pchlide and NADPH (Figs. 3D, 4A, B). Tyr94, Thr145, Thr147 and Asn149 are all located in close proximity to the propionate side-chain of the Pchlide molecule at the C17 position, whereas Thr230 is located at a key position near to the diphosphate chain joining the adenosine and the nicotinamide ribose groups of NADPH. However, although the Ser189 and His236 residues are positioned relatively near to the Pchlide molecule and have previously been implicated in Pchlide binding from previous homology models [27], the new structural model shows that this is unlikely as their distance from Pchlide is too great to allow any direct interactions with the pigment (Fig. 4B).

### 3.2 Steady-state Characterization of Active Site POR Variants

The new structural model of POR, in addition to previous structural homology models [5], [27], [28], [29], [30], have highlighted a number of residues that may be important for catalytic activity. These residues, which can potentially interact with the NADPH coenzyme [40], [41], the Pchlide substrate or are in close proximity to the binding site of both substrates [5], [27], [28], [29], [30], were altered by site-directed mutagenesis and their catalytic activity initially determined under steady-state conditions (Table 1). Although many of the variants retained close to wild-type levels of activity, several showed a significant reduction in catalytic activity. In particular, mutations to Asn39, Asn90, Thr145, Thr147 and Asn149 had a major impact on steady-state activity, whereas replacement of the Thr230 residues with a bulkier Phe residue showed a marked decrease in activity, in contrast to the limited effects caused by replacement with Ala or Ser.

**Table 1:** The steady-state kinetic parameters, coenzyme and substrate binding constants and rates of hydride and proton transfer for wild-type POR and variant enzymes. All rates and binding constants were measured at 25 °C. The rates of hydride and proton transfer were measured from the average of at least five time dependent absorption measurements by laser excitation of similar levels of ternary enzyme-substrate complex and by following absorbance changes at 696 nm. The amplitude of the absorbance change at 696 nm is also shown.

Enzyme	*k*_cat_ (s^− 1^)	*K*_d_^NADPH^ (nM)	*K*_d_^Pchlide^ (μM)	*k*_hydride_ (× 10^6^ s^− 1^)	*k*_proton_ (× 10^4^ s^− 1^)	ΔmAbs 696 nm
Wild-type	0.17 ± 0.002	21 ± 1	5.6 ± 0.6	2.21 ± 0.06	2.72 ± 0.04	102 ± 1
S16C	0.16 ± 0.001	325 ± 90	3.5 ± 0.5	2.11 ± 0.03	2.68 ± 0.03	61 ± 3
G19A	0.16 ± 0.001	182 ± 31	3.5 ± 0.4	2.08 ± 0.07	2.67 ± 0.02	92 ± 2
R38V	0.16 ± 0.001	411 ± 76	5.4 ± 0.6	2.12 ± 0.03	2.69 ± 0.01	54 ± 4
N39V	0.02 ± 0.001	23 ± 6	28.9 ± 1.4	1.98 ± 0.02	2.71 ± 0.03	10 ± 0.4
K42A	0.16 ± 0.001	230 ± 81	4.5 ± 0.4	2.14 ± 0.09	2.67 ± 0.04	85 ± 3
N90A	0.07 ± 0.002	94 ± 7	21.4 ± 0.6	2.28 ± 0.04	3.17 ± 0.04	31 ± 2
Y94F	0.17 ± 0.003	63 ± 4	11.8 ± 0.7	2.23 ± 0.02	3.11 ± 0.01	80 ± 1
T145A	0.01 ± 0.001	33 ± 2	39.7 ± 2.1	n. d.	n. d.	2 ± 0.2
T147S	0.02 ± 0.003	60 ± 3	6.5 ± 0.7	n. d.	n. d.	2 ± 0.2
T147F	0.01 ± 0.003	117 ± 3	10.3 ± 0.6	n. d.	n. d.	1 ± 0.1
N149V	0.01 ± 0.003	55 ± 2	19.2 ± 1.2	n. d.	n. d.	2 ± 0.2
S189A	0.16 ± 0.001	38 ± 16	4.9 ± 0.4	2.15 ± 0.07	2.69 ± 0.01	69 ± 4
T230A	0.16 ± 0.001	181 ± 16	11.9 ± 1.4	1.63 ± 0.07	2.26 ± 0.03	51 ± 3
T230S	0.16 ± 0.001	48 ± 17	4.6 ± 0.5	1.71 ± 0.06	2.38 ± 0.06	60 ± 1
T230F	0.01 ± 0.001	436 ± 87	44.9 ± 3.5	n. d.	n. d.	2 ± 0.2
H236A	0.10 ± 0.004	30 ± 11	9.4 ± 0.6	2.18 ± 0.03	2.72 ± 0.01	33 ± 1

In order to provide a more in-depth rationale for this variation in catalytic activities, the ability of all variant enzymes to bind the coenzyme NADPH and Pchlide was measured (Table 1). The dissociation constant for NADPH was determined by measuring the FRET signal from Trp residue(s) in the protein to the bound NADPH coenzyme [38]. Several variants, including S16C, G19A, R38V, K42A, T147F, T230A and T230F, had a significantly reduced affinity for NADPH compared to the wild-type enzyme, indicating a role for the targeted residues in coenzyme binding. The binding of Pchlide to the enzyme–NADPH complex has been monitored by measuring the red-shift in the absorbance maximum of the Pchlide molecule upon forming a ternary POR–Pchlide–NADPH ternary complex (Table 1) [24]. Most of the variant POR enzymes exhibited only minor changes in the Pchlide binding properties. However, exceptions to this were the N39V, N90A, T145A, N149V and T230F variants, which showed ~ 5–10 fold reduction in the affinity of the Pchlide substrate.

### 3.3 Laser Excitation Measurements of the Site-directed Mutants

**Fig. 5:**
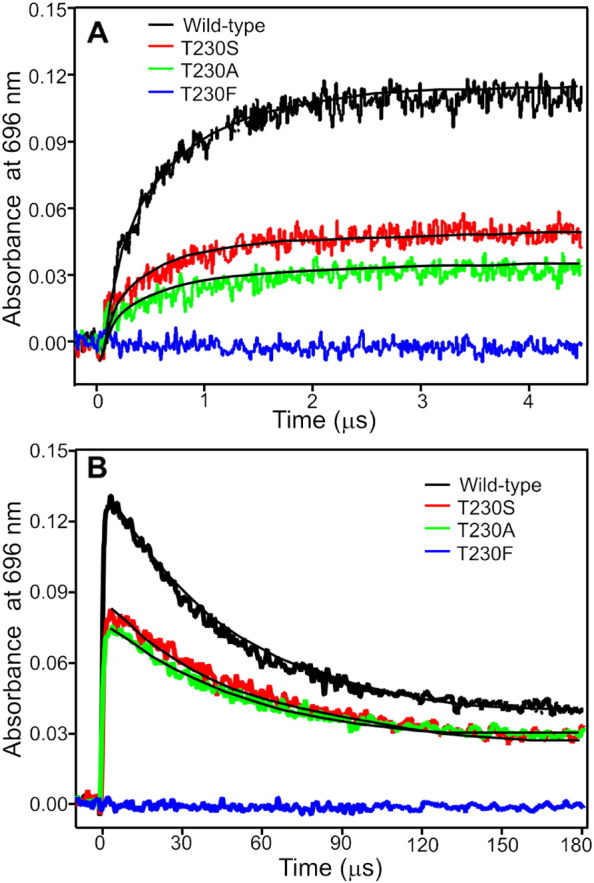
Hydride and proton transfer transients of wild-type and Thr230 variant POR enzymes. Typical laser transients showing the absorbance change at 696 nm for the wild-type POR and the Thr230 variants. The formation of the absorbance band at 696 nm represents hydride transfer and the disappearance of the 696 nm band represents proton transfer [15]. The transients were measured using an equal ternary complex concentration based on the Pchlide binding constant for each enzyme. The experimental procedure is described in the materials and method section.

Laser photoexcitation measurements have been used to obtain information on the excited state and single-turnover kinetics of intermediate formation for wild-type POR and the variant enzymes. The formation of a broad absorbance band at 696 nm represents the hydride transfer chemistry from NADPH and the disappearance of this 696 nm band represents the proton transfer step to the C18 position to form the final Chlide product [10], [11]. Hence, the kinetics and relative yield of formation/decay of the absorbance band at 696 was measured in dark-assembled enzyme-substrate ternary complex samples after excitation with a 6 ns laser pulse. For those variants whose reaction could be accurately measured there were only minor differences in the rates of hydride and proton transfer (Table 1). More significantly, many of the mutations resulted in a major loss of the amplitude of the absorbance signal at 696 nm upon photoexcitation (Table 1), suggesting that the quantum yield or photochemical efficiency of the hydride transfer step is impaired in those variants (example transients are shown in Fig. 3). In some cases, notably the N149V, T145A, T147S and T230F variants, the photochemical efficiency was so low (Table 1) that it was not possible to measure accurate rates for the hydride and proton transfer steps, indicating a potentially important role for these residues in photochemistry. Indeed, replacement of the Thr230 residue with a bulkier Phe residue in the T230F variant reduced the photochemical efficiency significantly compared to the T230A and T230S variants (Fig. 5).

**Fig. 6:**
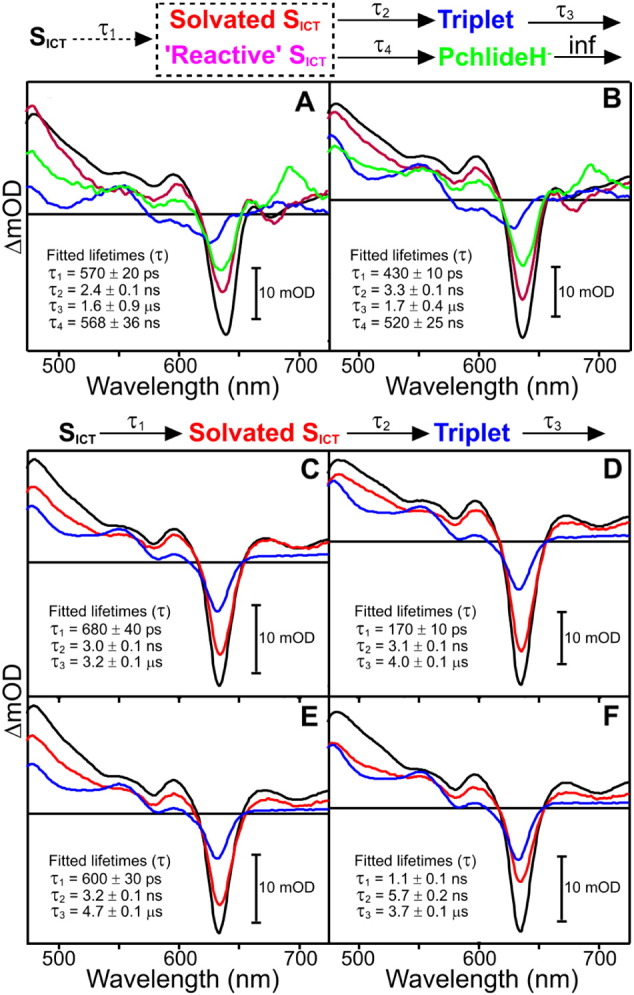
Species associated difference spectra (SADS) resulting from a global analysis of the time-resolved visible data for wild type and variant POR–Pchlide–NADPH ternary complexes after excitation at 450 nm. The data for wild type (A) and N39V (B) were fitted as described in the Supporting Information to a branched model (shown above the panel), where 60% of the ICT state is converted to the solvated ICT state along the ‘non-catalytic’ pathway and 40% is converted to a ‘reactive’ ICT state along the ‘catalytic’ pathway [18]. The data for T145A (C), T147S (D), N149V (E) and T230F (F) were fitted to the sequential model (shown above the panel) as described in Supporting Information. Kinetic traces showing fits at selected energies are shown in Fig. S9.

To further explore this potential role in photochemistry the excited state dynamics were investigated by pump-probe transient absorption spectroscopy for 5 POR variants that showed an impaired photochemical efficiency and compared to those obtained for Pchlide only and a wild-type POR–Pchlide–NADPH ternary complex. The time-resolved absorption difference spectra from these measurements were then modeled using global analysis to yield species associated difference spectra (SADS). For clarity only the SADS are shown in the main manuscript, whereas the raw time-resolved data (Figs. S3–S9), and kinetic traces showing fits at selected probe wavelengths (Fig. S10) can be found in the supporting information. In the wild-type POR ternary enzyme-substrate complex the observed transient spectral changes can be fitted to a branched model along ‘catalytic’ and ‘non-catalytic’ pathways after the formation of an intramolecular charge transfer state (S_ICT_) as described previously [18]. In this model the formation of the hydride transfer intermediate, with the characteristic absorbance band at 696 nm, is observed with a lifetime of approximately 500 ns (Fig. 6A), similar to that obtained in the earlier laser flash photolysis measurements. The excited state dynamics observed for the N39V variant could also be fitted to the same branched model with similar spectral features and lifetimes (Fig. 6B), suggesting that photochemistry proceeds in an identical way to the wild type enzyme. In contrast, the hydride transfer intermediate is completely absent in the N149V, T145A, T147S and T230F variants (Fig. 6C-F), confirming that photochemistry is impaired in these variants. In all of these cases the SADS required to model the transient spectral data appear to be very similar to free Pchlide (Fig. S10) and could be fitted to 3 sequentially evolving exponential functions. This represents a simple, linear decay pathway for the excited state dynamics in these variants, involving the solvation of the ICT state, followed by decay of the S1/ICT excited state into a long-lived triplet state on the ns timescale. The triplet state then relaxes back to the ground state on the μs timescale [18].

## 4 Discussion

It is now known that light-induced interactions between the protein and the pigment occur within the lifetime of the Pchlide excited state and are essential for the subsequent chemical steps, involving sequential hydride and proton transfer reactions, on the microsecond timescale [8], [15], [18], [21], [22]. However, the lack of any crystal structure for POR has made it difficult to understand the structural elements and active site residues that are required for this unique light-driven catalysis. Consequently, a number of residues located in the proposed substrate binding sites of POR have now been altered and their role in the reaction mechanism of POR investigated by static and time-resolved spectroscopic measurements. Moreover, the results have been rationalized in terms of active site structure based on a new structural model of the enzyme.

The majority of SDR enzymes contain a highly conserved nucleotide-binding domain, termed the Rossmann fold [40], [41], [42], [43], [44], which is characterized by a TGxxxGxG motif (TGASSGVG in *T. elongatus* POR). Mutations to Ser16 and Gly19 in this region were found to result in a lower affinity for NADPH, although the catalytic activity remained unaffected. Similarly, changes to Arg38 and Lys42 also reduced NADPH affinity and the close proximity of these residues to the 2′-phosphate and hydroxyl groups of the adenosine ribose of the coenzyme in the structural model support their important role in defining the coenzyme specificity in the SDR family [45]. In addition, Thr230 may also be important for the optimal positioning of the NADPH coenzyme in the active site as it appears to be located at a key position near to the diphosphate chain joining the adenosine and the nicotinamide ribose groups. Although the affinity for NADPH was reduced for both the T230 A and T230S variants the effect was much greater when a bulkier Phe residue was placed at this position.

In terms of Pchlide binding Asn39, Asn90, Thr145 and Asn149 appear to be important as mutations to these residues reduce both the catalytic efficiency of the enzyme and the affinity of the substrate. In the majority of SDR enzymes there is a catalytic tetrad of active site residues that are essential for activity, consisting of Asn-Ser-Tyr-Lys [44], [46]. It has been suggested that the Ser stabilizes the substrate and the Tyr acts as a proton donor, whereas the Lys lowers the p*Ka* of the Tyr-OH to promote proton transfer and the Asn residue provides important interactions with the Lys to maintain its position in the active site and promote the proton relay mechanism [44], [46]. The importance of the active site Tyr and Lys residues in POR catalysis have already been reported in previous studies [11]. However, in POR from *T. elongatus* the Ser residue from the catalytic tetrad is replaced by a Thr (Thr145), which is located in close proximity to the catalytic Tyr and Lys and also to the NADPH coenzyme in the structural model. Hence, changes to this residue may significantly influence any interactions between the Tyr, Lys, NADPH and Pchlide substrate. The Asn from the catalytic tetrad is Asn90 in POR, which is likely to play a similar role to other SDR enzymes by stabilizing the active site geometry through interactions with the ribose hydroxyl group of the nicotinamide and the active site Lys [44], [46]. However, the present work has revealed that changes to two other residues, Ser189 and His236, which had been suggested to be important for Pchlide binding based on previous homology models [27], only have a minimal effect on substrate binding and catalytic activity. Although the new structural model of POR described in the present work is similar to previous homology models [5], [27], [28], [29], [30], the Pchlide binding site differs significantly and indicates that the proposed role of His236 in chelating the central Mg ion of Pchlide is unlikely as there is no possibility for direct interaction with Mg^2 +^. In addition, the previous suggestion that Ser189 may be important in the correct positioning of the Pchlide towards the *pro*-S face of NADPH [27] is also unlikely as the S189 A variant retained wild-type levels of substrate binding and catalytic activity. The crystal structure of the unrelated light-independent Pchlide reductase shows that there is no requirement for the Pchlide substrate to have a direct Mg^2 +^ coordination with amino acid residues in the active site [6], [7]. Hence, based on the new model, His236 together with other aromatic residues such as Phe233 and Phe237, may be involved in Pchlide binding by providing a pi stacking interaction with the substrate (Fig. 4). However, His236 is replaced by serine in some homologous POR enzymes, which indicates that this residue is not an absolute necessity. Moreover, Pro96 and Leu232 are close to the Mg^2 +^ in the new model and it is possible that these residues are also involved either in a direct or indirect coordination with the central metal ion (Fig. 4).

Importantly, the present work highlights how minor changes to the architecture of the active site can have a profound effect on the efficiency of photochemistry. Previous studies have shown that the photochemistry is driven by excited state interactions between the active site Tyr and the carboxyl group of the propionate side chain at the C17 position of Pchlide, which pulls electron density away from the C17–C18 double bond to form a ‘reactive’ charge transfer state [18]. This creates an electron-deficient site across the double bond, which triggers the subsequent transfer of the negatively charged hydride from NADPH [18]. Several of the active site variants, including T145A, T147S, T147F, N149V and T230F exhibit impaired photochemical behaviour, implying a role for these residues in the formation of the excited state ‘reactive’ charge transfer state. Based on the structural model the Thr147 and Asn149 residues are in close enough proximity to interact directly with the propionate side-chain at the C17 position of Pchlide. As this region of the Pchlide molecule is essential for photochemistry [18] it is likely that both of these residues are essential for the excited state interactions that create the ‘reactive’ charge transfer state. As discussed above, the close proximity of Thr145 to the catalytic Tyr and Lys and the NADPH coenzyme may mean that any changes to this residue can significantly affect any excited state interactions between the Tyr, Lys, NADPH and Pchlide substrate. Although the Thr230 is unlikely to directly participate in any excited state interactions it may still play an important role in maintaining the active site geometry to allow photochemistry to proceed efficiently as replacement with a bulkier Phe residue abolishes the photochemical step.

## Concluding remarks

We have now highlighted a number of key residues in the active site of POR that are important for coenzyme and substrate binding, as well as the excited state processes required for catalysis. The role of these residues have been verified by a new structural model for *T. elongatus* POR, which supports all of the findings from the binding, multiple turnover and single turnover laser studies on the active site variants. In the absence of any crystal structure this work will provide the basis for future functional studies of this key light-activated enzyme.

## Appendix A Supplementary data

Supplementary material. Image 2
