# Supplementary material for: Multiple active site residues are important for photochemical efficiency in the light-activated enzyme protochlorophyllide oxidoreductase (POR)
Source: J Photochem Photobiol B. 2016 Aug;161:236–43. doi: 10.1016/j.jphotobiol.2016.05.029 (PMC4970445; doi:10.1016/j.jphotobiol.2016.05.029)
Supplement: Supplementary file 1 — Supplementary material. [file mmc1.docx]

**Multiple active site residues are important for photochemical efficiency in the light-activated enzyme protochlorophyllide oxidoreductase (POR).**

**Binuraj R. K. Menon, Samantha J. O. Hardman, Nigel S. Scrutton*, Derren J. Heyes***

^1^Centre for Synthetic Biology of Fine and Speciality Chemicals, Manchester Institute of Biotechnology, School of Chemistry, The University of Manchester, Manchester, M1 7DN, UK.

**Table S1**. The amino acid sequence of *T. elongatus* POR and SDR enzyme templates.

| > *T. elongatus* POR  MSDQPRPTVIITGASSGVGLYATKALANRGWHVIMACRNLEKAEQAAKNLQIPPEAYTILHLDLSSLASVRGFVESFRALNRPLRALVCNAAVYYPLLKEPIYSVDGYEITVATNHLGHFLLINLLLEDLKNSPESDKRLVILGTVTANRKELGGKIPIPAPPDLGNLEGFEKGFKKPIAMINGKPFKSGKAYKDSKLCNMLTARELHRRFHESTGIVFNSLYPGCVADTPLFRHHFPLFQKLFPLFQKKITGGYVSQELAGERVAMVVADPEFRQSGVHWSWGNRQKEGRKAFVQELSAEASDEQKARRLWELSEKLVGLA |
| --- |
| >3rd5  TGWTAADLPSFAQRTVVITGANSGLGAVTARELARRGATVIMAVRDTRKGEAAARTMAGQVEVRELDLQDLSSVRRFADGVSGADVLINNAGIMAVPYALTVDGFESQIGTNHLGHFALTNLLLPRLTDRVVTVSSMAHWPGRINLEDLNWRSRRYSPWLAYSQSKLANLLFTSELQRRLTAAGSPLRALAAHPGYSHTNLATDADFGARQTLYAASQDLPGDSFVGPRFGYLGRTQPVGRSRRAKDAGMAAALWALSEQLTKTEFPL |
| >2hrb  SRVALVTGANRGIGLAIARELCRQFSGDVVLTARDVARGQAAVQQLQAEGLSPRFHQLDIDDLQSIRALRDFLRKEYGGLNVLVNNAAVAFKSDDPMPFDIKAEMTLKTNFFATRNMCNELLPIMKPHGRVVNISSLQCLRAFENCSEDLQERFHSETLTEGDLVDLMKKFVEDTKNEVHEREGWPNSPYGVSKLGVTVLSRILARRLDEKRKADRILVNACCPGPVKTDMDGKDSIRTVEEGAETPVYLALLPPDATEPQGQLVHDKVVQNW |
| >2b4q  MHPYFSLAGRIALVTGGSRGIGQMIAQGLLEAGARVFICARDAEACADTATRLSAYGDCQAIPADLSSEAGARRLAQALGELSARLDILVNNAGTSWGAALESYPVSGWEKVMQLNVTSVFSCIQQLLPLLRRSASAENPARVINIGSVAGISAMGEQAYAYGPSKAALHQLSRMLAKELVGEHINVNVIAPGRFPSRMTRHIANDPQALEADSASIPMGRWGRPEEMAALAISLAGTAGAYMTGNVIPIDGGFHL |
| >4jig > 4JIG  SSDRKAVLITGASRGIGRATAVLAAERGWDVGINYARDAAAAELTAQAVRDAGGRACIVAGDVANEADVVAMFDTVAAAFGRLDALVNNAGIVAPSMPLADMPVDRLRRMFDTNVLGAYLCAREAARRLSTDRGGRGGAIVNVSSIASRLGSPNEYVDYAGSKGAVDSLTIGLAKELGPHGVRVNAVRPGLIETRLGAQTPLGRAGEAQEVAEAIVWLLGDTASYTTGALLDVGGGR |
| >4dc1  QDSEVALVTGATSGIGLEIARRLGKEGLRVFVCARGEEGLRTTLKELREAGVEADGRTCDVRSVPEIEALVAAVVERYGPVDVLVNNAGRPGGGATAELADELWLDVVETNLTGVFRVTKQVLKAGGMLERGTGRIVNIASTGGKQGVVHAAPYSASKHGVVGFTKALGLELARTGITVNAVCPGFVETPMAASVREHFSDIWEVSTEEAFDRITARVPIGRYVQPSEVAEMVAYLIGPGAAAVTAQALNVCGGLGNY |
| >1n5d  SSNTRVALVTGANKGIGFAIVRDLCRQFAGDVVLTARDVARGQAAVKQLQAEGLSPRFHQLDIIDLQSIRALCDFLRKEYGGLDVLVNNAAIAFQLDNPTPFHIQAELTMKTNFMGTRNVCTELLPLIKPQGRVVNVSSTEGVRALNECSPELQQKFKSETITEEELVGLMNKFVEDTKNGVHRKEGWSDSTYGVTKIGVSVLSRIYARKLREQRAGDKILLNACCPGWVRTDMGGPKAPKSPEVGAETPVYLALLPSDAEGPHGQFVTDKKVVEWGVPPESYPWVNA |
| >4hwk  GLGRAVCLLTGASRGFGRTLAPLLASLLSPGSVLVLSARNDEALRQLEAELGAERSGLRVVRVPADLGAEAGLQQLLGALRELPRPKGLQRLLLINNAGSLGDVSKGFVDLSDSTQVNNYWALNLTSMLCLTSSVLKAFPDSPGLNRTVVNISSLCALQPFKGWALYCAGKAARDMLFQVLALEEPNVRVLNYAPGPLDTDMQQLARETSVDPDMRKGLQELKAKGKLVDCKVSAQKLLSLLEKDEFKSGAHVDFYD |
| >1wma  SGIHVALVTGGNKGIGLAIVRDLCRLFSGDVVLTARDVTRGQAAVQQLQAEGLSPRFHQLDIDDLQSIRALRDFLRKEYGGLDVLVNNAGIAFKVADPTPFHIQAEVTMKTNFFGTRDVCTELLPLIKPQGRVVNVSSIMSVRALKSCSPELQQKFRSETITEEELVGLMNKFVEDTKKGVHQKEGWPSSAYGVTKIGVTVLSRIHARKLSEQRKGDKILLNACCPGWVRTDMAGPKATKSPEEGAETPVYLALLPPDAEGPHGQFVSEKRVEQW |
| >3tox  SRLEGKIAIVTGASSGIGRAAALLFAREGAKVVVTARNGNALAELTDEIAGGGGEAAALAGDVGDEALHEALVELAVRRFGGLDTAFNNAGALGAMGEISSLSVEGWRETLDTNLTSAFLAAKYQVPAIAALGGGSLTFTSSFVGHTAGFAGVAPYAASKAGLIGLVQALAVELGARGIRVNALLPGGTDTPANFANLPGAAPETRGFVEGLHALKRIARPEEIAEAALYLASDGASFVTGAALLADGGASVTK |
| >3v8b  PSPVALITGAGSGIGRATALALAADGVTVGALGRTRTEVEEVADEIVGAGGQAIALEADVSDELQMRNAVRDLVLKFGHLDIVVANAGINGVWAPIDDLKPFEWDETIAVNLRGTFLTLHLTVPYLKQRGGGAIVVVSSINGTRTFTTPGATAYTATKAAQVAIVQQLALELGKHHIRVNAVCPGAIETNISDNTKLRHEEETAIPVEWPKGQVPITDGQPGRSEDVAELIRFLVSERARHVTGSPVWIDGGQGLLR |
| >1oaa  ADGLGCAVCVLTGASRGFGRALAPQLARLLSPGSVMLVSARSESMLRQLKEELGAQQPDLKVVLAAADLGTEAGVQRLLSAVRELPRPEGLQRLLLINNAATLGDVSKGFLNVNDLAEVNNYWALNLTSMLCLTSGTLNAFQDSPGLSKTVVNISSLCALQPYKGWGLYCAGKAARDMLYQVLAAEEPSVRVLSYAPGPLDNDMQQLARETSKDPELRSKLQKLKSDGALVDCGTSAQKLLGLLQKDTFQSGAHVDFYD |
| >3tzq  AELENKVAIITGACGGIGLETSRVLARAGARVVLADLPETDLAGAAASVGRGAVHHVVDLTNEVSVRALIDFTIDTFGRLDIVDNNAAHSDPADMLVTQMTVDVWDDTFTVNARGTMLMCKYAIPRLISAGGGAIVNISSATAHAAYDMSTAYACTKAAIETLTRYVATQYGRHGVRCNAIAPGLVRTPRLEPQPIVDIFATHHLAGRIGEPHEIAELVCFLASDRAAFITGQVIAADSGLLAHLPGLPQIRASVAEL |
| >4trr  MSNLNGKTAVVTGAASGIGKEIALELAKAGAAVAIADLNQDGANAVADEINKAGGKAIGVAMDVTNEEAVNTGIDKVAEAFGSVDILVSNAGIQIVNPIENYSFADWKKMQAIHVDGAFLTTKAALKHMYKDDRGGVVIYMGSVHSHEASPLKSAYVTAKHGLLGLARVLAKEGAKHNVRSHVVCPGFVRTPLVDKQIPEQISEEEVIKKVMLGNTVDGVFTTVQDVAQTVLFLSAFPSAALTGQSFIVSHGWFMQ |
| >1vl8  FDLRGRVALVTGGSRGLGFGIAQGLAEAGCSVVVASRNLEEASEAAQKLTEKYGVETMAFRCDVSNYEEVKKLLEAVKEKFGKLDTVVNAAGINRRHPAEEFPLDEFRQVIEVNLFGTYYVCREAFSLLRESDNPSIINIGSLTVEEVTMPNISAYAASKGGVASLTKALAKEWGRYGIRVNVIAPGWYRTKMTEAVFSDPEKLDYMLKRIPLGRTGVPEDLKGVAVFLASEEAKYVTGQIIFVDGGWTAN |
| >1xg5  ARPGMERWRDRLALVTGASGGIGAAVARALVQQGLKVVGCARTVGNIEELAAECKSAGYPGTLIPYRCDLSNEEDILSMFSAIRSQHSGVDICINNAGLARPDTLLSGSTSGWKDMFNVNVLALSICTREAYQSMKERNVDDGHIININSMSGHRVLPLSVTHFYSATKYAVTALTEGLRQELREAQTHIRATCISPGVVETQFAFKLHDKDPEKAAATYECLKPEDVAEAVIYVLSTPAHIQIGDIQMRPTGS |

**Table S2.** The 15 SDR enzyme selected based on the higher sequence identity with *T. elongatus* POR. PDB identity number and sequence identity with *T. elongatus* POR are shown. The SWISS-MODEL homology server was used for the selection of SDR enzymes as described in the materials and methods section.

| No | Title | Organism | PDB ID | Resolution  Å | Sequence identity | Reference |
| --- | --- | --- | --- | --- | --- | --- |
| 1 | Putative uncharacterized protein | *Mycobacterium partuberculosis* | 3RD5 | 1.5 | 29.89 | Baugh *et al*, 2015 |
| 2 | Human carbonyl reductase | *Homo sapiens* | 2HRB | 1.9 | 23.90 |  |
| 3 | Rhamnolipids biosynthesis 3-oxoacyl-[acyl-carrier-protein] reductase | *Pseudomonas aeruginosa* | 2B4Q | 2.3 | 22.45 | Miller *et al*, 2006 |
| 4 | Dehydrogenase | *Burkholderia cenocepacia* | 4JIG | 1.8 | 22.41 |  |
| 5 | Ketoacyl reductase | *Streptomyces coelicolor* | 4DC1 | 2.8 | 21.81 | Javidpour *et al*, 2013 |
| 6 | 20β-hydroxysteroid dehydrogenase | *Sus scrofa. Pig.* | 1N5D | 2.3 | 21.79 | Ghosh *et al*, 2001 |
| 7 | Sepiapterin reductase | *Homo sapiens* | 4HWK | 2.4 | 21.76 |  |
| 8 | Carbonyl reductase | *Homo sapiens* | 1WMA | 1.2 | 21.65 | Tanaka *et al*, 2005 |
| 9 | Short chain dehydrogenase | *Sinorhizobium meliloti* | 3TOX | 1.9 | 21.31 |  |
| 10 | 3-oxoacyl-[acyl-carrier protein] reductase | *Sinorhizobium meliloti.* | 3V8B | 2.7 | 21.22 |  |
| 11 | Sepiapterin reductase | *Mus musculus. House mouse.* | 1OAA | 1.2 | 21.16 | Auerbach *et al*, 1997 |
| 12 | Short-chain type dehydrogenase/reductase | *Mycobacterium marinum* | 3TZQ | 3.5 | 20.85 | Baugh *et al*, 2015 |
| 13 | D-beta-hydroxybutyrate dehydrogenase | *Burkholderia cenocepacia* | 4TRR | 1.9 | 20.33 |  |
| 14 | Gluconate 5-dehydrogenase | *Thermotoga maritima* | 1VL8 | 2.0 | 20.25 |  |
| 15 | Human putative dehydrogenase MGC4172 | *Homo sapiens* | 1XG5 | 1.5 | 19.26 |  |

**Table S3**. The primers that were used for site directed mutagenesis in POR.

| No | Primer Name | Primer |
| --- | --- | --- |
| 1 | S16C forward primer | 5’-C ATA CAA TCC GAC TCC **GCA** GGA TGC ACC CGT AAT-3’ |
| 2 | S16C reverse primer | 5’-ATT ACG GGT GCA TCC **TGC** GGA GTC GGA TTG TAT G-3’ |
| 3 | G19A forward primer | 5’-CTT GGT AGC ATA CAA **TGC** GAC TCC CGA GGA TGC-3’ |
| 4 | G19A reverse primer | 5’-GCA TCC TCG GGA GTC **GCA** TTG TAT GCT ACC AAG-3’ |
| 5 | R38V forward primer | 5’-CTC TGC TTT TTC AAG ATT **GAC** GCA GGC CAT TAT AAC GTG-3’ |
| 6 | R38V reverse primer | 5’-CAC GTT ATA ATG GCC TGC **GTC** AAT CTT GAA AAA GCA GAG-3’ |
| 7 | N39V forward primer | 5’-TTG CTC TGC TTT TTC AAG **AAC** GCG GCA GGC CAT TAT AAC-3’ |
| 8 | N39V reverse primer | 5’-GTT ATA ATG GCC TGC CGC **GTT** CTT GAA AAA GCA GAG CAA-3’ |
| 9 | K42A forward primer | 5’-GC GGC TTG CTC TGC **TGC** TTC AAG AAT GCG GC-3’ |
| 10 | K42A reverse primer | 5’-GC CGC ATT CTT GAA **GCA** GCA GAG CAA GCC GC-3’ |
| 11 | N90A forward primer | 5’-ATA ATA GAC AGC GGC **AGC** GCA GAC AAG GGA CG-3’ |
| 12 | N90A reverse primer | 5’-CGT GCC CTT GTC TGC **GCT** GCC GCT GTC TAT TAT-3’ |
| 13 | Y94F forward primer | 5’-C CTT GAG CAG GGG ATA **AAA** GAC AGC GGC ATT GCA G-3’ |
| 14 | Y94F reverse primer | 5’-C TGC AAT GCC GCT GTC **TTT** TAT CCC CTG CTC AAG G-3’ |
| 15 | T145A forward primer | 5’-GCG GTT GGC TGT CAC **TGC** GCC GAG AAT CAC CAA-3’ |
| 16 | T145A reverse primer | 5’-TTG GTG ATT CTC GGC **GCA** GTG ACA GCC AAC CGC-3’ |
| 17 | T147S forward primer | 5’-TTC TTT GCG GTT GGC TGA CAC **TGT** GCC GAG AAT-3’ |
| 18 | T147S reverse primer | 5’-ATT CTC GGC ACA GTG **TCA** GCC AAC CGC AAA GAA-3’ |
| 19 | T147F forward primer | 5’-TTC TTT GCG GTT GGC TGA CAC **TTC** GCC GAG AAT-3’ |
| 20 | T147F reverse primer | 5’-ATT CTC GGC ACA GTG **GAA** GCC AAC CGC AAA GAA-3’ |
| 21 | N149V forward primer | 5’- GGC ACA GTG ACA GCC **GTT** CGC AAA GAA CTC GGC G-3’ |
| 22 | N149V reverse primer | 5’- C GCC GAG TTC TTT GCG **AAC** GGC TGT CAC TGT GCC -3’ |
| 23 | S189A forward primer | 5’-TTT GTA GGC CTT GCC **CGC** CTT GAA GGG CTT ACC-3’ |
| 24 | S189A reverse primer | 5’-GGT AAG CCC TTC AAG **GCG** GGC AAG GCC TAC AAA-3’ |
| 25 | T230A forward primer | 5’-GT TGT GTG GCC GAC **GCA** CCC CTG TTT CGC CA-3’ |
| 26 | T230A reverse primer | 5’-TG GCG AAA CAG GGG **TGC** GTC GGC CAC ACA AC-3’ |
| 27 | T230S forward primer | 5’-GGT TGT GTG GCC GAC **TCA** CCC CTG TTT CGC CA-3’ |
| 28 | T230S reverse primer | 5’-TG GCG AAA CAG GGG **TGA** GTC GGC CAC ACA ACC-3’ |
| 29 | T230F forward primer | 5’-GT TGT GTG GCC GAC **TTC** CCC CTG TTT CGC CA-3’ |
| 30 | T230F reverse primer | 5’-TG GCG AAA CAG GGG **GAA** GTC GGC CAC ACA AC-3’ |
| 31 | H236A forward primer | 5’-T GAA ACA GGG GGA AGG CGT **GGC** GAA ACA GGG G-3’ |
| 32 | H236A reverse primer | 5’-C CCC TGT TTC **GCC** ACG CCT TCC CCC TGT TTC A-3’ |

**
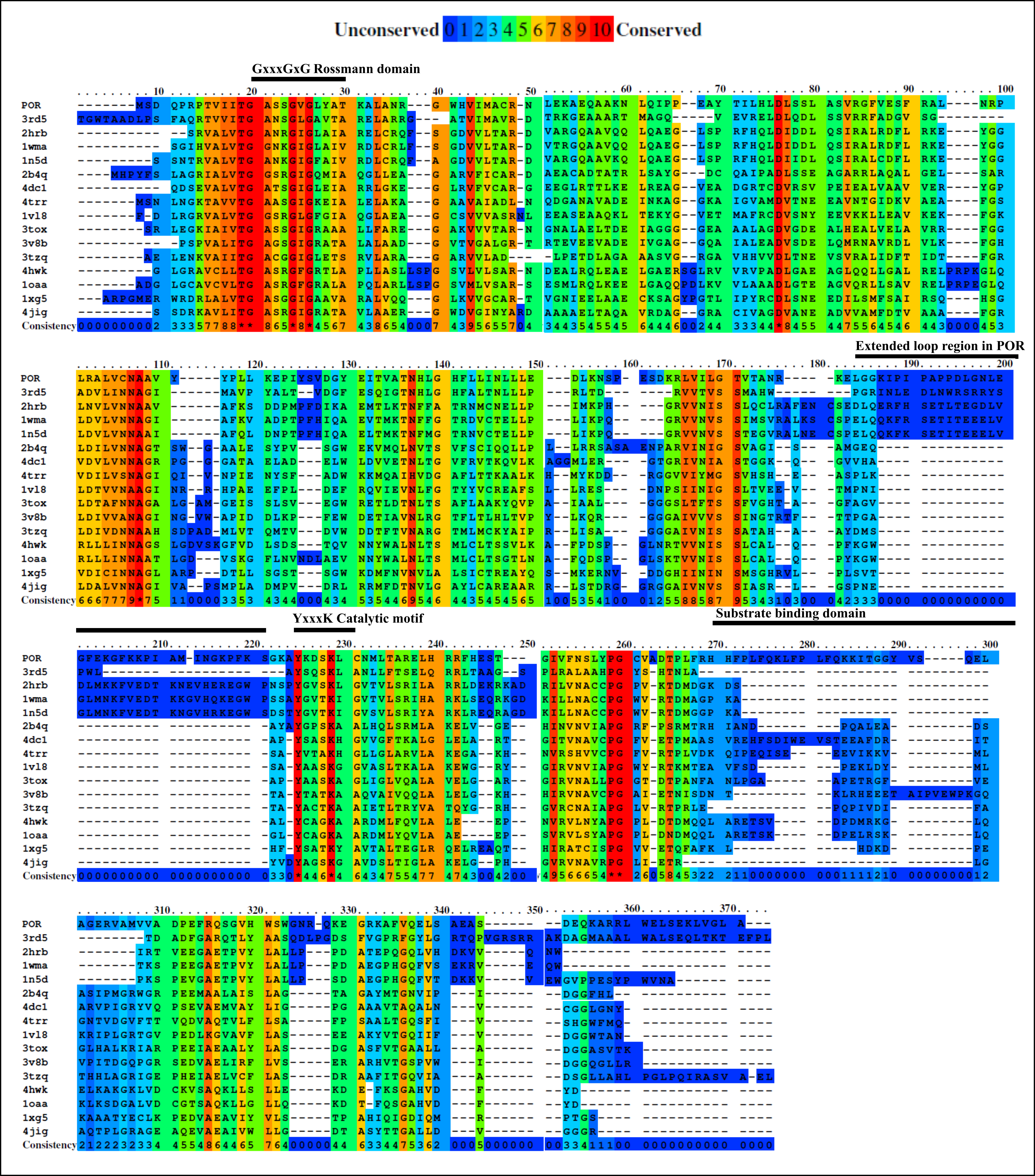
**

**Figure S1. The sequence alignment of selected SDR structural templates with POR.** The protein sequence of T. elongatus POR is aligned with the best 15 SDR enzymes of known structures in PDB that shares a higher sequence identity with *T. elongatus* POR is shown. PRALINE; protein multiple sequence alignment web server tool (by Centre for Integrative Bioinformatics, Vrije Universiteit Amsterdam, Netherlands) was used for the alignment of protein sequences.


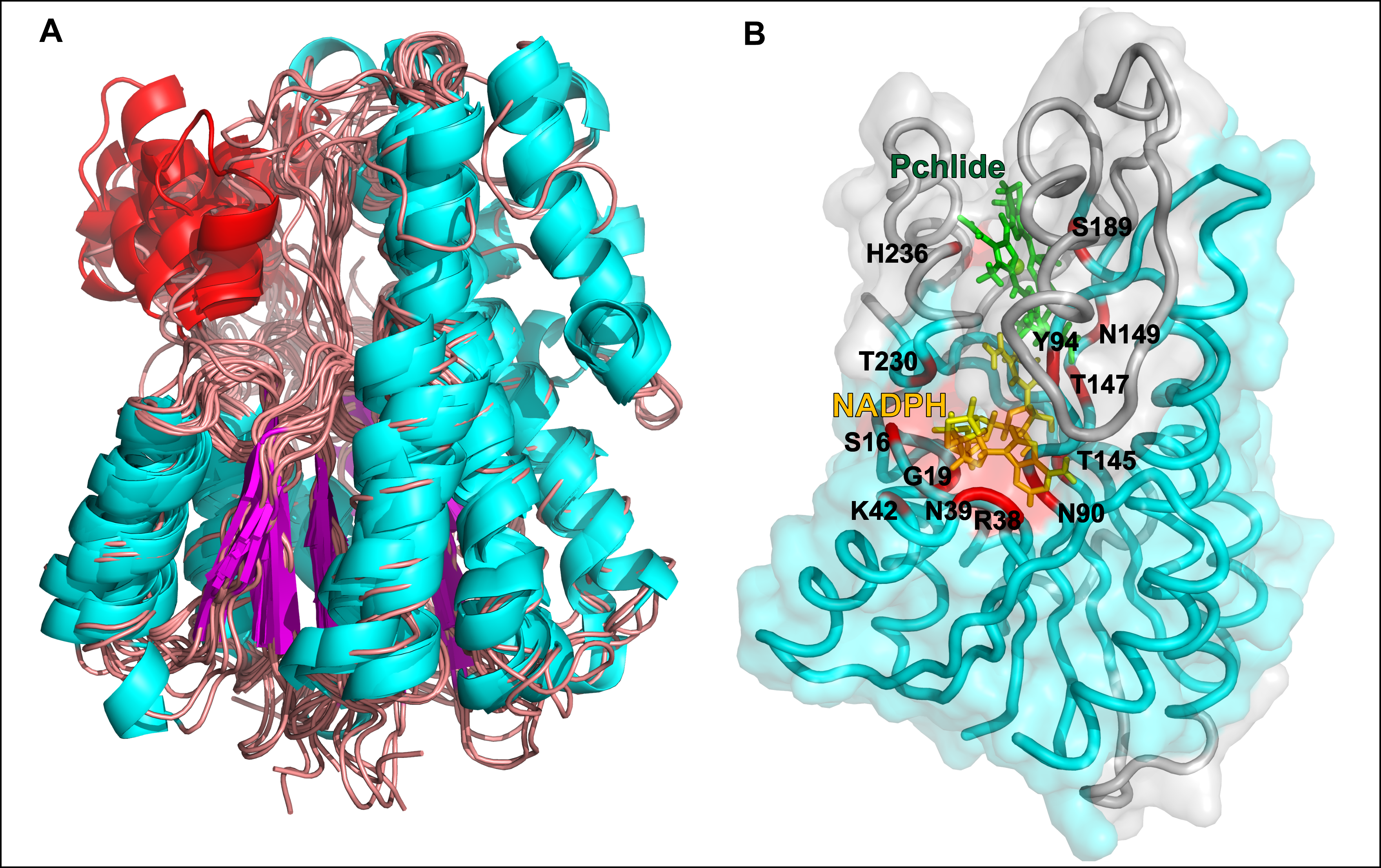


**Figure S2. A.** The structural super positioning of the 15 structurally characterized SDR enzymes that share the highest sequence identity with *T. elongatus* POR. The substrate binding regions which are non-aligned with each other are shown in *red* colour. **B.** The relative position of the mutated residues in the derived structural model for *T. elongatus* POR. The Mustang-MR structural sieving server was used for the multiple structural alignments of these SDR enzymes.

**



**

**Figure S3.** Time-resolved transient absorption spectroscopy data for wild-type POR-Pchlide-NADPH ternary complex after photoexcitation with a laser pulse centred at ~450 nm. Time-resolved difference spectra were recorded between 1 ps and 2 µs as described in the Methods section.

**



**

**Figure S4.** Time-resolved transient absorption spectroscopy data for N39V POR-Pchlide-NADPH ternary complex after photoexcitation with a laser pulse centred at ~450 nm. Time-resolved difference spectra were recorded between 1 ps and 2 µs as described in the Methods section.

**



**

**Figure S5.** Time-resolved transient absorption spectroscopy data for T145A POR-Pchlide-NADPH ternary complex after photoexcitation with a laser pulse centred at ~450 nm. Time-resolved difference spectra were recorded between 1 ps and 2 µs as described in the Methods section.

**



**

**Figure S6.** Time-resolved transient absorption spectroscopy data for T147S POR-Pchlide-NADPH ternary complex after photoexcitation with a laser pulse centred at ~450 nm. Time-resolved difference spectra were recorded between 1 ps and 2 µs as described in the Methods section.

**



**

**Figure S7.** Time-resolved transient absorption spectroscopy data for N149V POR-Pchlide-NADPH ternary complex after photoexcitation with a laser pulse centred at ~450 nm. Time-resolved difference spectra were recorded between 1 ps and 2 µs as described in the Methods section.

**



**

**Figure S8.** Time-resolved transient absorption spectroscopy data for T230F POR-Pchlide-NADPH ternary complex after photoexcitation with a laser pulse centred at ~450 nm. Time-resolved difference spectra were recorded between 1 ps and 2 µs as described in the Methods section.





**Figure S9.** Kinetic traces at selected wavelengths with corresponding fits resulting from a global analysis of the time-resolved visible data for the POR-Pchlide-NADPH ternary complexes of wild-type (A), and variants N39V(B), T145A(C), T147S(D), N149V(E), T230F(F). The data were fitted as described in the Methods section.


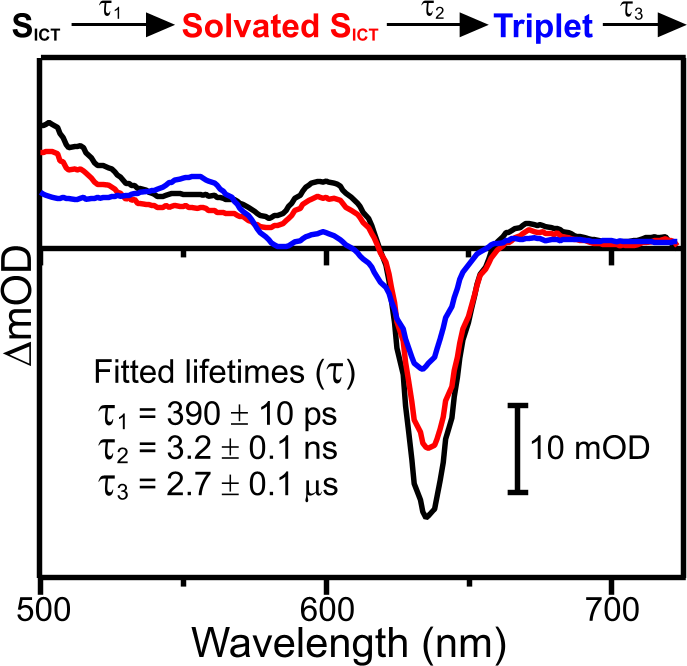


**Figure S10.** Species associated difference spectra (SADS) resulting from a global analysis of the time-resolved visible data for Pchlide only after excitation at 450 nm. The data were fitted to the sequential model (shown above the panel) as described in Supporting Information.

**Supplemental references**:

[1] L. Baugh, I. Phan, D.W. Begley, M.C. Clifton, B. Armour, D.M. Dranow, B.M. Taylor, M.M. Muruthi, J. Abendroth, J.W. Fairman, D. Fox, S.H. Dieterich, B.L. Staker, A.S. Gardberg, R. Choi, S.N. Hewitt, A.J. Napuli, J. Myers, L.K. Barrett, Y. Zhang, M. Ferrell, E. Mundt, K. Thompkins, N. Tran, S. Lyons-Abbott, A. Abramov, A. Sekar, D. Serbzhinskiy, D. Lorimer, G.W. Buchko, R. Stacy, L.J. Stewart, T.E. Edwards, W.C. Van Voorhis, P.J. Myler, Increasing the structural coverage of tuberculosis drug targets, Tuberculosis, 95 (2015) 142-148.

[2] D.J. Miller, Y.M. Zhang, C.O. Rock, S.W. White, Structure of RhlG, an essential beta-ketoacyl reductase in the rhamnolipid Biosynthetic pathway of Pseudomonas aeruginosa, Journal of Biological Chemistry, 281 (2006) 18025-18032.

[3] P. Javidpour, J. Bruegger, S. Srithahan, T.P. Korman, M.P. Crump, J. Crosby, M.D. Burkart, S.C. Tsai, The Determinants of Activity and Specificity in Actinorhodin Type II Polyketide Ketoreductase, Chemistry & Biology, 20 (2013) 1225-1234.

[4] D. Ghosh, M. Sawicki, V. Pletnev, M. Erman, S. Ohno, S. Nakajin, W.L. Duax, Porcine carbonyl reductase - Structural basis for a functional monomer in short chain dehydrogenases/reductases, Journal of Biological Chemistry, 276 (2001) 18457-18463.

[5] M. Tanaka, R. Bateman, D. Rauh, E. Vaisberg, S. Ramachandani, C. Zhang, K.C. Hansen, A.L. Burlingame, J.K. Trautman, K.M. Shokat, C.L. Adams, An unbiased cell morphology-based screen for new, biologically active small molecules, Plos Biology, 3 (2005) 764-776.

[6] G. Auerbach, A. Herrmann, M. Gutlich, M. Fischer, U. Jacob, A. Bacher, R. Huber, The 1.25 angstrom crystal structure of sepiapterin reductase reveals its binding mode to pterins and brain neurotransmitters, Embo Journal, 16 (1997) 7219-7230.
